# The effect of on- and off-road cycle paths on cycling to work: A study using administrative data

**DOI:** 10.23889/ijpds.v8i2.2263

**Published:** 2023-09-14

**Authors:** Laurie Berrie, Zhiqiang Feng, David Rice, Tom Clemens, Lee Williamson, Chris Dibben

**Affiliations:** 1 Ulster University, Belfast, United Kingdom; 2 National Records of Scotland, Edinburgh, United Kingdom

## Objectives

Promoting cycling as a means of transport is now on multiple policy agendas, including health, transport and climate change. In this work, we use administrative data to explore whether cycle paths, particularly off road compared to on road, might increase the proportion of people choosing to cycle to work.

## Methods

Our work used data from Edinburgh and Glasgow, Scotland in 2011 at the time of the population census. We limited our data to those aged 16–74 with a current job on the night of the census. Cycle route data were made computer readable from cycle route maps from the time of the census. We looked at the characteristics of those who cycled to work and how proximity to cycle path differed between groups. We used logistic regression to model cycle commuting against distance from home to the nearest cycle path adjusting for confounders identified using a Directed Acyclic Graph.

## Results

In this paper we will show how the propensity to cycle commute differs across population characteristics such as sex, age and the National Statistics Socio-economic classification and how the interaction of these factors affects propensity to cycle commute. We also show that living closer to on- and off-road cycle paths increases the likelihood of cycle commuting.

## Conclusion

Understanding interventions, such as developing on- and off-road cycle paths, that could increase the number and diversity of people cycle commuting (especially if they move from ‘passive’ to ‘active’ modes of travel) has the potential to reduce carbon emissions, congestion and air pollution, increase physical activity and therefore improve health.

